# Supplementary material for: Design principles for energy transfer in the photosystem II supercomplex from kinetic transition networks
Source: Nat Commun. 2024 Oct 9;15:8763. doi: 10.1038/s41467-024-53138-z (PMC11464844; doi:10.1038/s41467-024-53138-z)
Supplement: Supplementary file 1 — Supplementary Information: Design Principles for Energy Transfer in the Photosystem II Supercomplex from Kinetic Transition Networks [file 41467_2024_53138_MOESM1_ESM.pdf]

Supplementary Information: Design Principles for Energy Transfer in the Photosystem II Supercomplex from Kinetic Transition Networks

Shiun-Jr Yang[1,2,3], David J. Wales[4*], Esmae J. Woods[4,5], Graham R. Fleming[1,2,3*]

[1]Department of Chemistry, University of California, Berkeley, Berkeley, 94720, CA, USA.
[2]Molecular Biophysics and Integrated Bioimaging Division, Lawrence Berkeley National Laboratory, Berkeley, 94720, CA, USA.
[3]Kavli Energy Nanoscience Institute at Berkeley, Berkeley, 94720, CA, USA.
[4]Yusuf Hamied Department of Chemistry, University of Cambridge, Lensfield Road,Cambridge CB2 1EW,UK.
[4]Cavendish Laboratory, Department of Physics, University of Cambridge, Lensfield Road,Cambridge CB3 0HE,UK.

*Corresponding author(s). E-mail(s): dw34@cam.ac.uk; grfleming@lbl.gov;

Suppl. Note 1. Modeling Details

The kinetic model for the EET network in the $C_2S_2M_2$-type PSII-SC was generated based on the methods applied by Bennett et al. [1] and Leonardo et al. [2] The intra-protein Hamiltonians, including site energies and intra-protein couplings, were obtained from the literature [1, 3–7] (references are cited in the footnotes of Table 1). Due to the lack of semi-empirical Hamiltonians for CP26 and CP24, in the current PSII-SC model, they are replaced by the Hamiltonians of CP29 and LHCII, respectively, with absent Chls deleted. The parameters used in the simulation are the same as in ref [2] and are listed in Table 1 and Table 2. Since the calculation only focuses on EET dynamics at 300K, the dephasing term mentioned in ref [2] was omitted in the current simulation. The same approach was used in ref [1].

Different spectral densities are used for different subunits to ensure consistency with the Hamiltonians reported in the literature. For the subunits of the PSII core, the spectral density is defined as [1, 4]:

$$\chi''(\omega) = (\pi\hbar)\frac{S_0}{s_1 + s_2}\sum_{i=1,2}\frac{s_i\omega^5}{7!2\omega_i^4}e^{-\sqrt{\frac{\omega}{\omega_i}}} \tag{1}$$

where S_0, s_1, s_2, ω_1, and ω_2 are the parameters listed in Table 2. For the peripheral antennae, including LHCII, CP29, CP26, and CP24, the spectral density is defined as [6, 7]:

$$\chi''(\omega) = 2\lambda_0\frac{\omega\Gamma_0}{\omega^2 + \Gamma_0^2} \tag{2}$$

where λ_0 and Γ_0 are the parameters listed in Table 2. In addition, the contribution from under-damped modes are also included for the peripheral antennae, which has the following form [1, 7]:

$$\chi''_{\mathrm{vib}}(\omega) = \sum_{j=1}^{N_{\mathrm{vib}}} 2S_j\omega_j^3\frac{\omega\Gamma_{\mathrm{vib}}}{(\omega_j^2 - \omega^2)^2 + \omega^2\Gamma_{\mathrm{vib}}^2} \tag{3}$$

where S_j, ω_j, and Γ_{vib} are the parameters for each vibration mode. The values of these parameters can be found in ref [1] and ref [6].

As mentioned in the Methods, the definition of domains allows fast intra-domain EET and slow inter-domain EET. Bennett et al. have shown that it is a valid assumption that thermal equilibrium between the states within the same domain can be reached much faster than inter-domain EET. In the current work, we still obtain dynamics based on the full rate matrices that contain all exciton-to-exciton transfer rates. In addition, there are domains that are delocalized between multiple subunits in the model of Bennett et al., whereas no domains are delocalized in the model of Leonardo et al., which was calculated based on the cryo-EM structure of the C_2S_2-type PSII-SC (PDB:3JCU) [8]. In the current model, constructed based on the cryo-EM structure of the $C_2S_2M_2$-type PSII-SC (PDB:5XNL) [9], there are domains that are delocalized between different LHCII monomers (for both S-LHCII and M-LHCII) and between M-LHCII (C) and CP29. For delocalized domains, the subunit an exciton belongs to is defined as the one whose Chls have the most contribution to the exciton.

Suppl. Table 1 EET simulation parameters.

	RC[1,9]		CP43[2]/CP47[3]	CP29/CP26[4]		LHCII/CP24[5]	
Pigment	Chl *a*	Pheo	Chl *a*	Chl *a*	Chl *b*	Chl *a*	Chl *b*
TDM[6]	4.4	3.5	4.4	3.74	3.18	4	3.4
σ_{inhom}[7]	200	200	180	90	108	80	96
SD type[8]	a	b	c	d	e	f	g

[1]Raszewski et al. [3, 4] (adapted by Bennett et al. [1])

[2]Müh et al. [5] (adapted by Bennett et al. [1])

[3]Raszewski et al. [4] (adapted by Bennett et al. [1])

[4]Mascoli et al. [6]

[5]Novoderezhkin et al. [7] (adapted by Bennett et al. [1])

[6]Transition dipole moment magnitude [unit: Debye]

[7]Inhomogeneous broadening width [unit: cm^{-1}]

[8]Spectral density type in Table 2

[9]σ_{inhom} for Chlz is 120 cm^{-1}. Other parameters are the same as Chl *a*.

Suppl. Table 2 Spectral density parameters (a detailed description can be found in SI Section Simulation Details).

	a	b	c	d	e	f	g
S_0	0.65	0.65	0.5				
s_1	0.8	0.8	0.8				
s_2	0.5	0.5	0.5				
ω_1 [cm^{-1}]	0.532	0.532	0.532				
ω_2 [cm^{-1}]	1.94	1.94	1.94				
λ_0 [cm^{-1}]				40	48	37	48
γ_0 [cm^{-1}]				40	40	30	30
UD BO[1]	No	No	No	Yes	Yes	Yes	Yes

[1]This row indicates whether under-damped Brownian oscillators were included in the spectral density.

Suppl. Note 2. Rates to Free Energies

To visualise the free energy landscape, we translate the rate matrix $\mathbf{K}$, into effective free energies [10, 11]. Each element K_{ij} is the transition rate from substate j to substate i. The effective free energies $f_s(T)$, for each state s, are defined in terms of the equilibrium occupation probabilities, π_s,

$$f_s(T) = -\mathrm{k_B} T \ln \pi_s, \tag{4}$$

where $\mathrm{k_B}$ is Boltzman's constant and T is the temperature. The effective free energy of the transition state that connects substate s to substate s' is $f^{\dagger}_{ss'}(T)$, which is chosen to reproduce the rate constants via the Eyring–Polanyi equation [12, 13]:

$$K_{s's} = \frac{\mathrm{k_B} T}{\mathrm{h}} \exp\left[-\frac{\left(f^{\dagger}_{ss'}(T) - f_s(T)\right)}{\mathrm{k_B} T}\right], \tag{5}$$

where h is Planck's constant. Rearranging gives,

$$f^{\dagger}_{ss'}(T) = f_{s'}(T) - k_B T \ln K_{ss'} + \mathrm{k_B} T \ln\left(\mathrm{k_B} T/\mathrm{h}\right), \tag{6}$$

$$= f_s(T) - \mathrm{k_B} T \ln K_{s's} + \mathrm{k_B} T \ln\left(\mathrm{k_B} T/h\right). \tag{7}$$

The $f_s(T)$ values were obtained by exploiting the detailed balance relations defined by the rate matrix entries and minimising a least squares problem using the `GMIN` global optimisation program [14].

Suppl. Note 3. Computing First Passage Time Distributions

Two alternative approaches were employed to calculate the first passage time distribution (FPT), and we summarise them here. First, we explain how eigendecomposition provides an analytical expression for the FPT. We then describe the complementary kinetic Monte Carlo (kMC) method, which samples individual trajectories from source to sink.

We consider the transition matrix $\mathbf{Q} = \mathbf{K} - \mathbf{D}$, where $\mathbf{D}$ is a diagonal matrix of escape rates, with elements $D_{jj} = \sum_\gamma K_{\gamma j}$. The kinetics are described by the linear master equation,

$$\frac{\mathrm{d}\mathbf{P}(\mathrm{t})}{\mathrm{d}t} = \mathbf{Q}\mathbf{P}(t), \tag{8}$$

where $\mathbf{P}(t)$ is the time-dependent vector of occupation probabilities for the substates. We are interested in the first passage time distributions, defined as the first hitting time for a trajectory to reach the sink $\mathcal{A}$, given an initial starting probability distribution. We set all the escape rates from the sink to zero, and define the substochastic matrix $\mathbf{Q}_\mathcal{S} = \mathbf{K}_\mathcal{S} - \mathbf{D}_\mathcal{S}$, where we have partitioned the state space into two disjoint sets, $\Omega = \mathcal{A} \cup \mathcal{S}$. Ω is the full state space, and $\mathcal{S}$ is the state space minus the sink. $\mathbf{Q}_\mathcal{S}$ is the subset of the full transition matrix $\mathbf{Q}$ containing the interstate transition rates within $\mathcal{S}$. $\mathbf{D}_\mathcal{S}$ is the corresponding subset of $\mathbf{D}$ including the escape rates to $\mathcal{A}$.

Eigendecomposition

The substochastic transition matrix can be decomposed into its constituent eigenmodes as,

$$\mathbf{Q}_\mathcal{S} = -\sum_{\ell}^{|\mathcal{S}|} \lambda_\ell \mathbf{w}_\ell^R \otimes \mathbf{w}_\ell^L, \tag{9}$$

where $\mathbf{w}_\ell^L$ and $\mathbf{w}_\ell^R$ are the left and right eigenvectors and $\otimes$ is the outer product. $\mathbf{w}_\ell^L$ is a row vector, and $\mathbf{w}_\ell^R$ is a column vector. All eigenvalues are real and negative, $-\lambda_\ell <$

0. Using the above decomposition, we can write the first passage time distribution as a summation over eigenmodes,

$$p(t) = \sum_{\ell=1}^{|\mathcal{S}|} \lambda_\ell e^{-\lambda_\ell t} \mathbf{1}_\mathcal{S} \left(\mathbf{w}_\ell^R \otimes \mathbf{w}_\ell^L \right) \mathbf{P}_\mathcal{S}(0). \tag{10}$$

Here, $\mathbf{1}_\mathcal{S}$ is a row vector of ones and $\mathbf{P}_\mathcal{S}(0)$ is the initial occupation probability in $\mathcal{S}$, at $t = 0$. It is useful to make the transformation $y = \ln t$, to produce the probability distribution $\mathcal{P}(y)$,

$$\mathcal{P}(y) = \sum_{\ell=1}^{|\mathcal{S}|} \lambda_\ell e^{y - \lambda_\ell e^y} \mathbf{1}_\mathcal{S} \left(\mathbf{w}_\ell^R \otimes \mathbf{w}_\ell^L \right) \mathbf{P}_\mathcal{S}(0). \tag{11}$$

As $p(t)$ and $\mathcal{P}(y)$ are normalised distributions, $\sum_\ell \mathbf{1}_\mathcal{S} \left(\mathbf{w}_\ell^R \otimes \mathbf{w}_\ell^L \right) \mathbf{P}_\mathcal{S}(0) = 1$.

Kinetic Monte Carlo

To analyse trajectory information in more detail, we have also run kMC simulations, which generate stochastic trajectories starting from the source and terminating at the sink [15–17]. Standard rejection-free kMC simulations work using two random numbers to generate the next transition and the associated timestep. If the trajectory currently lies in substate i, a random number r_1 is drawn uniformly with $r_1 \in (0, 1]$. The system is progressed to substate j, where,

$$\sum_{k=1}^{j-1} B_{ki} < r_1 \leq \sum_{k=1}^{j} B_{ki}. \tag{12}$$

The simulation clock time is incremented by $\Delta t = \tau_i \log r_2$, where r_2 is a second random number, also drawn uniformly with $r_2 \in (0, 1]$. $\mathbf{B}$ is the transition probability matrix with elements B_{ij} corresponding to the probability of transferring to substate i given a step is taken out of substate j. This process samples trajectories according

to the master equation. Using kMC simulations to compute FPT distributions enables trajectories to be assigned to particular time windows, which facilitates the dwell time distribution analysis.

Suppl. Note 4. NPQ Simulation

The NPQ simulation was carried out by adding a sink in one subunit in each of the monomer. For example, to understand the photoprotection ability of CP29, we place a quencher (the sink) in both CP29 of the PSII-SC. The sinks are connected to a group of Chls that are suggested to be the quenching sites upon activation of NPQ. In particular, two groups of Chls are tested: C610-C612 [18] and C602-C603 [19]. The results presented in the main text are based on the group of C610-C612, but there are no significant difference between the results of the two groups, as shown in Table 3 and 4. The transfer rates from the Chls to the sink is set to be $(200\ \text{fs})^{-1}$, similar to the values reported in literature [20, 21]. Quenching probability of a subunit is evaluated by averaging the probability of reaching the sink before reaching the RCs upon excitation of each exciton states within the subunit.

Suppl. Table 3 Quenching probability of different quencher locations upon excitation in all subunits in the PSII-SC when the quenching site is C610-C612.

	Quencher Location: C610-C612								
Excitation	CP26	S-A	S-B	S-C	CP24	CP29	M-A	M-B	M-C
M-A	0.46	0.45	0.67	0.58	0.31	0.78	0.84	0.57	0.78
M-B	0.42	0.40	0.60	0.52	0.34	0.86	0.77	0.83	0.86
M-C	0.38	0.36	0.52	0.47	0.38	0.92	0.66	0.58	0.92
CP24	0.36	0.32	0.47	0.43	0.87	0.92	0.57	0.48	0.92
CP29	0.36	0.32	0.46	0.43	0.36	0.94	0.56	0.47	0.94
CP47	0.26	0.22	0.31	0.31	0.24	0.59	0.36	0.30	0.59
RC	0.02	0.01	0.02	0.02	0.01	0.02	0.02	0.01	0.02
CP43	0.71	0.32	0.34	0.55	0.11	0.29	0.28	0.19	0.29
CP26	0.95	0.36	0.37	0.58	0.12	0.30	0.30	0.20	0.30
S-A	0.68	0.77	0.64	0.76	0.19	0.47	0.50	0.32	0.47
S-B	0.58	0.62	0.84	0.74	0.23	0.58	0.65	0.41	0.58
S-C	0.62	0.55	0.60	0.84	0.19	0.48	0.49	0.32	0.48

Suppl. Table 4 Quenching probability of different quencher locations upon excitation in all subunits in the PSII-SC when the quenching site is C602-C603.

	Quencher Location: C602-C603								
Excitation	CP26	S-A	S-B	S-C	CP24	CP29	M-A	M-B	M-C
M-A	0.45	0.52	0.61	0.58	0.30	0.78	0.84	0.67	0.74
M-B	0.41	0.46	0.54	0.52	0.33	0.85	0.83	0.91	0.85
M-C	0.38	0.41	0.48	0.47	0.37	0.91	0.71	0.67	0.86
CP24	0.35	0.37	0.43	0.42	0.87	0.92	0.62	0.56	0.71
CP29	0.35	0.37	0.43	0.42	0.35	0.93	0.60	0.54	0.70
CP47	0.26	0.26	0.29	0.29	0.23	0.59	0.39	0.35	0.45
RC	0.02	0.01	0.02	0.02	0.01	0.02	0.02	0.01	0.02
CP43	0.71	0.38	0.37	0.44	0.11	0.28	0.26	0.21	0.25
CP26	0.94	0.42	0.40	0.47	0.12	0.30	0.28	0.23	0.26
S-A	0.67	0.84	0.72	0.75	0.18	0.46	0.45	0.37	0.42
S-B	0.57	0.72	0.87	0.76	0.22	0.57	0.58	0.47	0.53
S-C	0.62	0.64	0.65	0.80	0.18	0.48	0.45	0.37	0.43

Suppl. Note 5. FPT Distribution: Results from kMC and Eigendecomposition

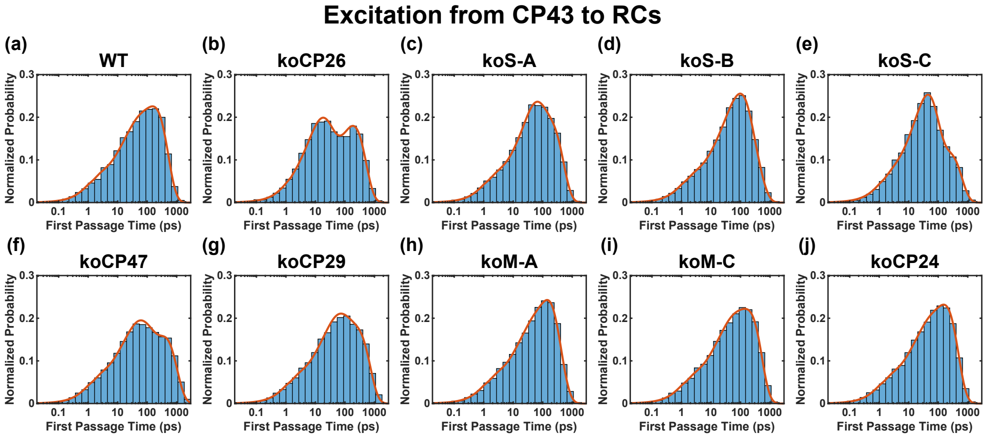

Suppl. Fig. 1 First passage time (FPT) distribution of excitations from CP43 to the RCs for (a) the WT, (b) koCP26, (c) koS-A, (d) koS-B, (e) koS-C, (f) koCP47 (g) koCP29, (h) koM-A, (i) koM-C, (j) koCP24. Orange lines are the FPT distributions from the analytical eigendecomposition of the transition matrix and the blue histograms are the FPT distributions from kMC trajectory counts. S-A: S-LHCII (A). S-B: S-LHCII (B). S-C: S-LHCII (C). M-A: M-LHCII (A). M-B: M-LHCII (B). M-C: M-LHCII (C). Labels of the PSII-SC subunits can be found in Figure 1b in the main text.

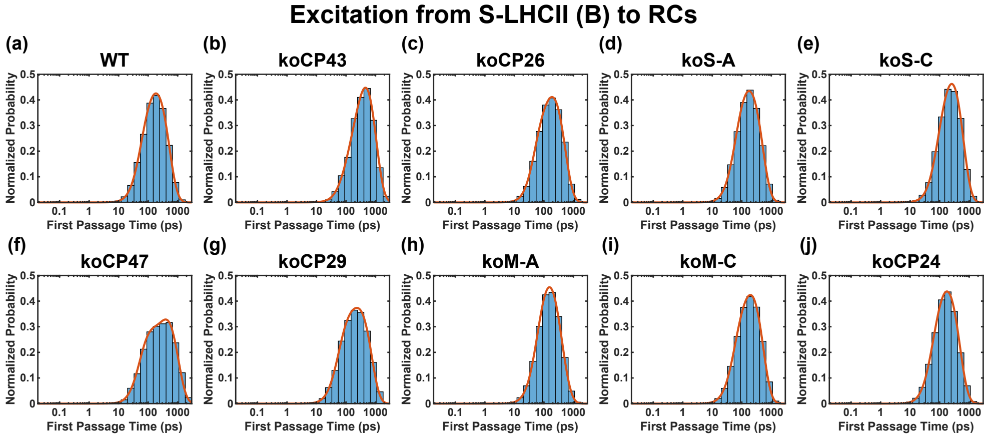

Suppl. Fig. 2 First passage time (FPT) distribution of excitations from S-LHCII (B) to the RCs for (a) the WT, (b) koCP43, (c) koCP26, (d) koS-A, (e) koS-C, (f) koCP47 (g) koCP29, (h) koM-A, (i) koM-C, (j) koCP24. Orange lines are the FPT distributions from analytical formulation and blue histograms are the FPT distributions from kMC trajectory counts. S-A: S-LHCII (A). S-B: S-LHCII (B). S-C: S-LHCII (C). M-A: M-LHCII (A). M-B: M-LHCII (B). M-C: M-LHCII (C). Labels of the PSII-SC subunits can be found in Figure 1b in the main text.

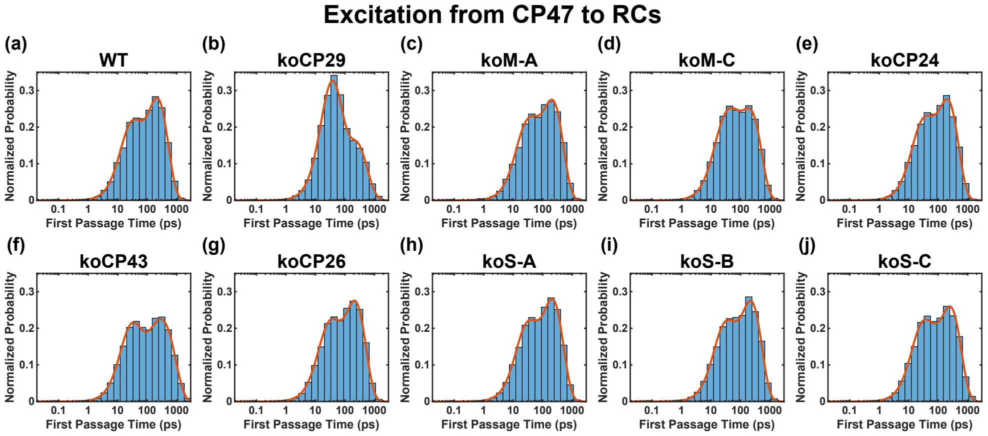

Suppl. Fig. 3 First passage time (FPT) distribution of excitations from CP47 to the RCs for (a) the WT, (b) koCP29, (c) koM-A, (d) koM-C, (e) koCP24, (f) koCP43 (g) koCP26, (h) koS-A, (i) koS-B, (j) koS-C. Orange lines are the FPT distributions from analytical formulation and blue histograms are the FPT distributions from kMC trajectory counts. S-A: S-LHCII (A). S-B: S-LHCII (B). S-C: S-LHCII (C). M-A: M-LHCII (A). M-B: M-LHCII (B). M-C: M-LHCII (C). Labels of the PSII-SC subunits can be found in Figure 1b in the main text.

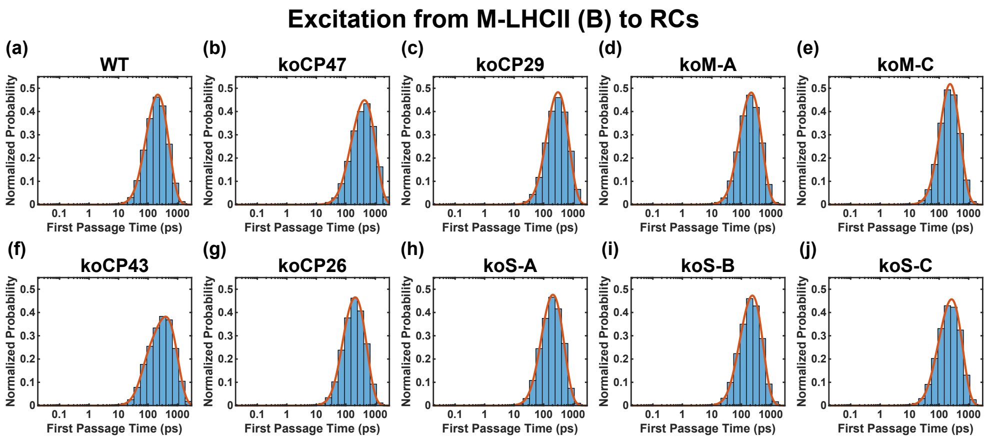

Suppl. Fig. 4 First passage time (FPT) distribution of excitations from CP47 to the RCs for (a) the WT, (b) koCP47, (c) koCP29, (d) koM-A, (e) koM-C, (f) koCP43 (g) koCP26, (h) koS-A, (i) koS-B, (j) koS-C. Orange lines are the FPT distributions from analytical formulation and blue histograms are the FPT distributions from kMC trajectory counts. S-A: S-LHCII (A). S-B: S-LHCII (B). S-C: S-LHCII (C). M-A: M-LHCII (A). M-B: M-LHCII (B). M-C: M-LHCII (C). Labels of the PSII-SC subunits can be found in Figure 1b in the main text.

Suppl. Note 6. Kinetic Analysis of Other Excitation Locations

Initial Excitations in S-LHCII (B)

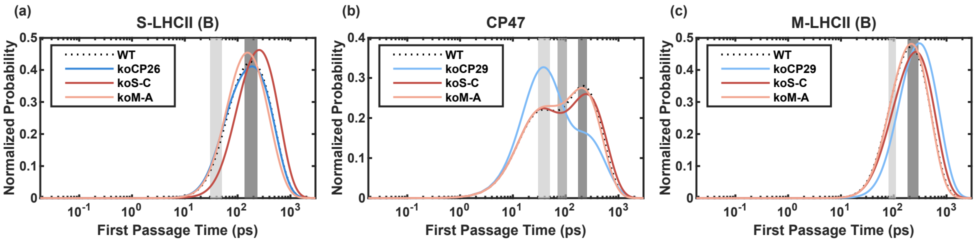

Suppl. Fig. 5 FPT distribution for the WT and selected mutants of PSII-SC starting from excitation in (a) S-LHCII (B), (b) CP47, (c) M-LHCII (B). The final state can be either RC. The shaded areas (light grey to dark grey) correspond to the FPT ranges discussed in this section and in Suppl. Fig 6-8.

Suppl. Fig. 5a shows the analytical FPT distributions of the WT and selected mutants for initial excitations in S-LHCII (B). The FPT distributions of the mutants are not very different from the WT. The FPT distribution of koS-C peaks at a slightly longer FPT while the FPT distribution of the M-LHCII (A) knockout (koM-A) has a peak at a shorter FPT relative to the WT.

For the trajectories with an FPT in the range 30 to 50 ps, the WT dwell time distribution (Suppl. Fig. 6a-d) shows that energy mostly stays within the D1 antennae before transferring to RC 1 from CP43 on this timescale. The dwell time distribution of koCP26 is similar to the WT. The only difference is the slightly longer dwell times at CP43 and S-LHCII (C). This result indicates that S-LHCII (C) can act as an alternative connection with the PSII core in addition to CP26. Consistent with this observation, the dwell times at CP26 and S-LHCII (A) are higher for koS-C than for the WT, providing more evidence for the presence of at least two pathways that

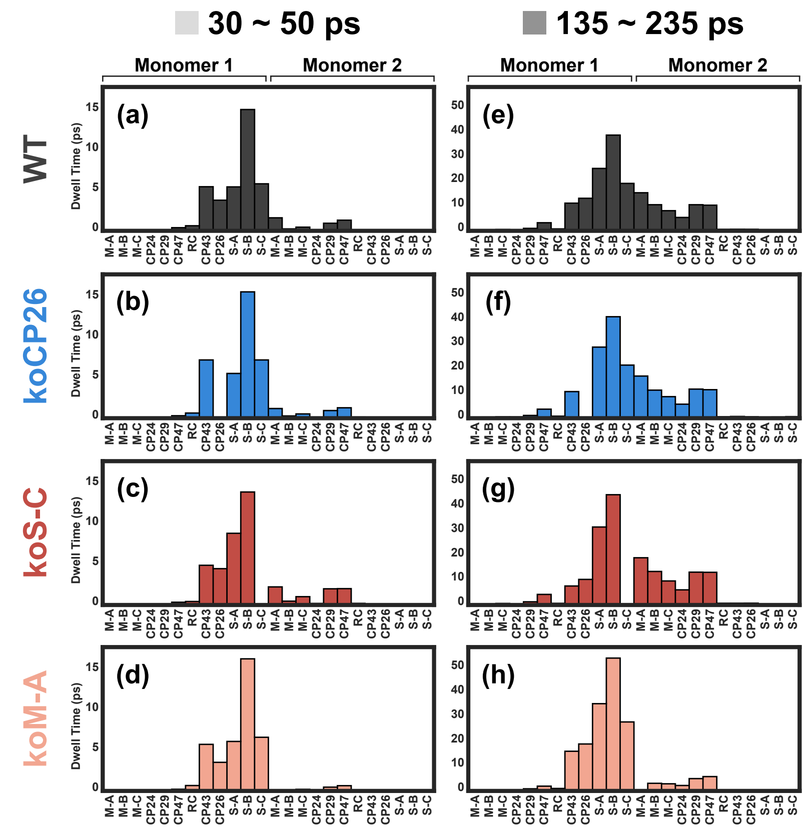

Suppl. Fig. 6 Dwell time distributions extracted from kMC trajectories for initial excitations in S-LHCII (B) in the FPT range of (a)-(d) 30 to 50 ps (Suppl. Fig. 5a, light grey), (e)-(h) 135 to 235 ps (Suppl. Fig. 5a, dark grey). The distributions from top to bottom are for the WT, koCP26, koS-C, and koM-A, respectively. In each panel, categories on the left are the subunits in Monomer 1 and categories on the right are those in Monomer 2.

transfer energy from the peripheral antenna system to the PSII core on this timescale—S-LHCII (C) or the combination of CP26 and S-LHCII (A). In the FPT distributions, a decrease in probability in this FPT range (Suppl. Fig. 5a, light grey) is observed for koS-C, but not for koCP26, suggesting that the S-LHCII (C) pathway dominates on this timescale. On the other hand, the removal of M-LHCII (A) greatly reduces the dwell times of the subunits on the D2 side of Monomer 2, indicating that M-LHCII (A) serves as the bridge between the subunits in S-LHCII and M-LHCII. However, the dwell time of CP29 and CP47 is nonzero, which means that there is an alternative

pathway connecting the two monomers. Based on the observation from CP43 excitation, S-LHCII (C) also acts as a bridge. Without M-LHCII (A) here, the other side of the connection must be CP29, as it is the only subunit close to S-LHCII (C).

For the trajectories with an FPT between 135 to 235 ps (Suppl. Fig. 6e-h), the dwell time distributions of koCP26 and koS-C are almost identical to the WT. This similarity arises because they can exchange roles in connecting the peripheral antenna system and the PSII core. The absence of any one of the these subunits does not have a significant effect on the ability to transfer energy to the RCs, especially on a longer timescale, where different pathways are more likely to be explored. We note that there is, however, an observable difference in the FPT distributions of koCP26 and koS-C compared to the distribution of the WT. The FPT distribution of koS-C has a peak at a longer FPT than the FPT distribution of the WT. This difference shows that the S-LHCII (C) pathway is faster than the CP26/S-LHCII (A) pathway. The most different dwell time distribution is observed for koM-A. The absence of M-LHCII (A) clearly blocks the most important pathways that connect the two monomers, as shown by the much shorter dwell times in all subunits in Monomer 2. Although S-LHCII (C) can transfer to CP29 on the D2 side, transferring to CP43 on the D1 side is clearly more favorable. This result, combined with the fact that the transfer from CP43 to the RC is faster than transfer from CP47 to the RC, leads to overall faster transfer to the RCs, causing the FPT distribution to peak at a shorter FPT than for the WT.

Initial Excitations in CP47

Suppl. Fig. 5b shows the analytical FPT distributions of the WT and selected mutants for initial excitations in CP47. The FPT distributions of all mutants are different from the FPT distribution of the WT. Unlike excitations in CP43, all the distributions have nearly identical peak positions. Only the peak heights vary. Among all the mutants, koCP29 exhibits the greatest difference.

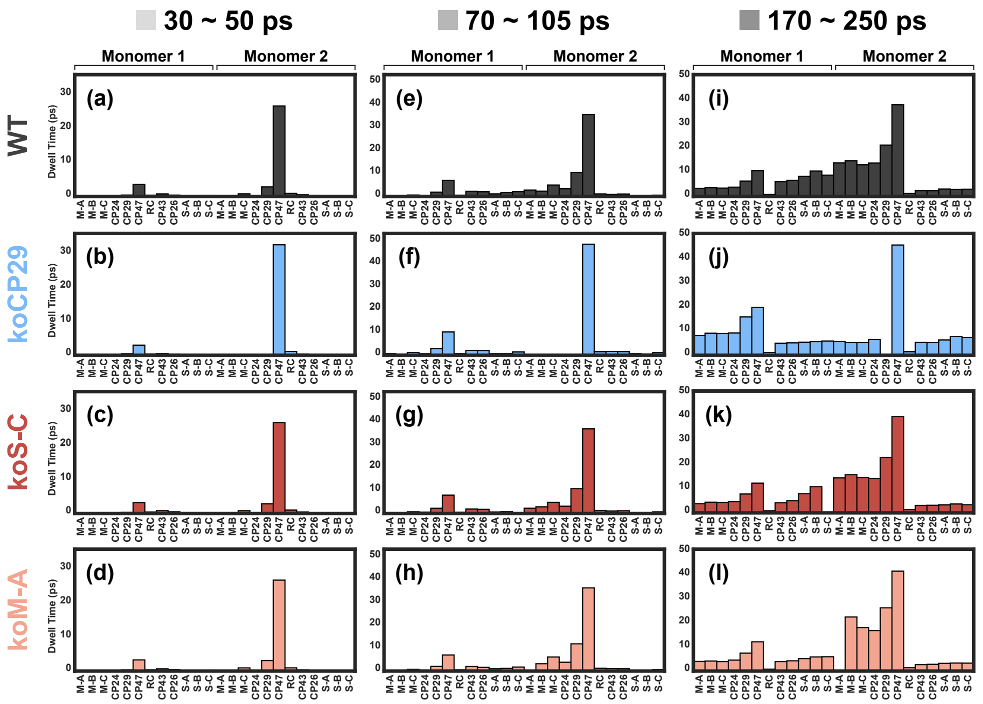

Suppl. Fig. 7 Dwell time distributions extracted from kMC trajectories for initial excitations in CP47 in the FPT range of (a)-(d) 30 to 50 ps (Suppl. Fig. 5b, light grey), (e)-(h) 70 to 105 ps (Suppl. Fig. 5b, grey), (i)-(l) 170 to 250 ps (Suppl. Fig. 5b, dark grey). The distributions from top to bottom are for the WT, koCP29, koS-C, and koM-A, respectively. In each panel, categories on the left are the subunits in Monomer 1 and categories on the right are those in Monomer 2.

For the trajectories with an FPT between 30 and 50 ps, the WT dwell time distribution (Suppl. Fig. 7a) shows that energy primarily stays in CP47 before entering RC 2. Besides staying in CP47 in Monomer 2, energy can also visit CP29 or the other CP47 in Monomer 1 and can barely travel beyond them. Even for CP29 and the CP47 in Monomer 1, dwell times are much shorter than for the CP47 in Monomer 2. The transfer between the two CP47 was discussed before in the PSII core complex [22]. Here, we also observe the same pathways that connect the left and the right side of the PSII dimer. Among all mutants, only koCP29 exhibits a different dwell time distribution from the WT. Clearly, the absence of CP29 blocks the only pathways leading energy out of the PSII core, leaving only the options of visiting RC 2 or the other

CP47 (in Monomer 1). This situation results in a much higher probability for energy to reach the RC on this timescale, reflected by the significantly higher peak at shorter FPTs in the FPT distribution of koCP29 (Suppl. Fig. 5c, blue). In addition, since the dwell time in CP47 is significantly longer than in other subunits in this FPT range, whether energy transfers from CP47 to CP29 or stays within CP47 does not cause a difference in the overall timescale, which is reflected by the identical peak positions of the FPT distributions of the mutants. For mutants other than koCP29, no difference from the WT is observed, as the subunits removed are not involved in the trajectories contributing to this FPT range.

For the trajectories with an FPT between 70 and 105 ps, the WT dwell time distribution (Suppl. Fig. 7e) exhibits non-zero dwell times for M-LHCII, S-LHCII, CP26 and CP43, in addition to the subunits mentioned above. Energy primarily stays on the left side of the PSII-SC. The only mutant that shows a different dwell time distribution is koCP29, where the pathways allowing the escape from CP47 to the periphery are removed. In this case, energy goes from the other CP47 (in Monomer 1) to the peripheral antennae on the right side of the PSII-SC on this timescale. Surprisingly, there are also non-zero dwell times for both CP43 and CP26 (in Monomer 1) even though the pathways to the left peripheral antennae are removed in the absence of CP29. This result shows that energy can actually be transferred from CP47 (of Monomer 2) to CP43 (of Monomer 1) without leaving the PSII core. One possible pathway is through $\mathrm{Chlz}_{\mathrm{D1}}$, which is located in the middle of the two subunits. Another possibility is that energy enters the RC from the other CP47 (of Monomer 1), and escapes to CP43 (Monomer 1). We note that, for all mutants, the dwell times of CP43 and CP26 are almost negligible, suggesting that the pathways involving these subunits are not relevant. The dwell time distributions of koS-C and koM-A are almost identical to the WT. This similarity again indicates that both S-LHCII (C) and M-LHCII (A) are complementary in their role of connecting the two monomers. However, the FPT

distribution of koS-C peaks at a slightly longer FPT, suggesting that S-LHCII (C) supports faster pathways. In contrast, the FPT distribution of koM-A peaks at a shorter FPT, suggesting that M-LHCII (A) is involved in slower pathways. This result is not surprising, as S-LHCII (C) is directly connected to CP29, while M-LHCII (A) is connected to S-LHCII (B). After transferring energy from M-LHCII (A) to S-LHCII (B), the fastest route still involves S-LHCII (C), as discussed in the previous section.

For the trajectories with an FPT in the range 170 and 250 ps, the dwell time distributions (Suppl. Fig. 7i-l) spread throughout the entire supercomplex, unlike the distributions of initial excitations in the D1 subunits, which mostly stay on the left side of the PSII-SC. For the WT, koS-C and koM-A, dwell times of the subunits on the right side of the PSII-SC are longer than those on the left side. The opposite behavior is observed for koCP29, which again, is due to the absence of pathways leading energy out of the core from the left side. The koCP29 FPT distribution (Suppl. Fig. 5, blue) has a much lower peak in this FPT range, indicating that energy has a higher probability to directly transfer into RC 2 from CP47 than to travel through the right side of the PSII-SC. In contrast to the shorter FPT range (70 to 105 ps), for which both koS-C and koM-A have similar dwell time distributions to the WT, the dwell time distribution of koM-A becomes more distinct while the distribution of koS-C remain similar to the WT. For koM-A, the dwell times of M-LHCII subunits are longer and the dwell times of S-LHCII subunits are shorter compared to the WT. This result shows that M-LHCII (A) is more important than S-LHCII (C) in connecting the two PSII monomers on this timescale. This difference arises because M-LHCII (A) has a good connection with S-LHCII (B), leading to longer pathways that explore the whole antenna system, i.e. S-LHCII and M-LHCII combined. S-LHCII (C), on the other hand, provides fast and straightforward pathways that lead energy directly to RC 1 from CP47 through CP29.

Initial Excitations in M-LHCII (B)

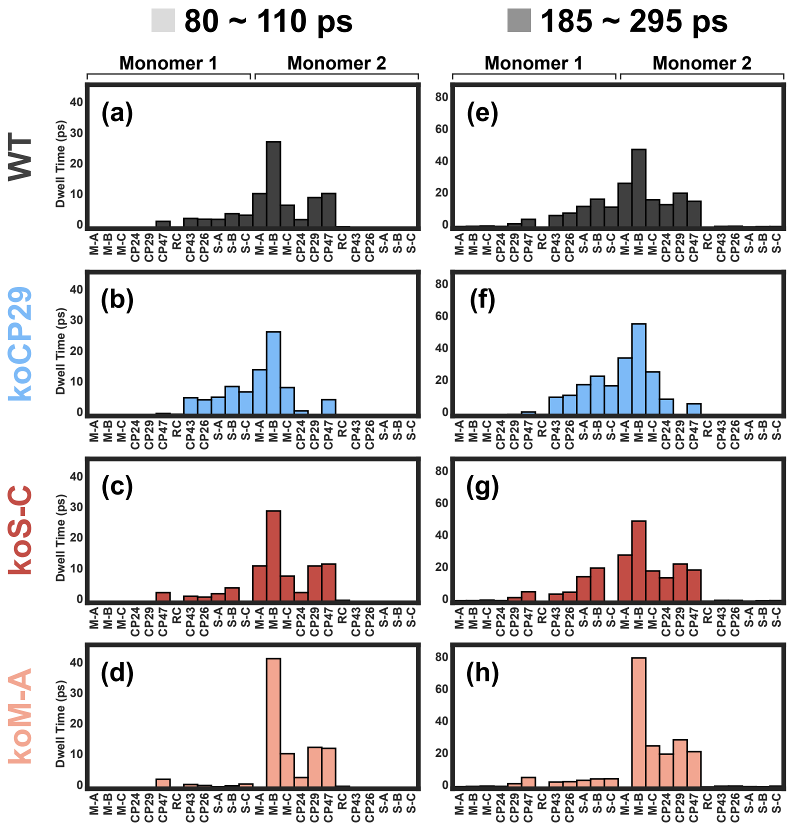

Suppl. Fig. 8 Dwell time distributions extracted from kMC trajectories for initial excitations in M-LHCII (B) in the FPT range of (a)-(d) 80 to 110 ps (Suppl. Fig. 5c, light grey), (e)-(h) 185 to 295 ps (Suppl. Fig. 5c, dark grey). The distributions from top to bottom are for the WT, koCP29, koS-C, and koM-A, respectively. In each panel, categories on the left are the subunits in Monomer 1 and categories on the right are those in Monomer 2.

Suppl. Fig. 5c shows the analytical FPT distributions of the WT and selected mutants for initial excitations localised in M-LHCII (B). The FPT distribution for this initial excitation condition is the least affected by the absence of subunits among the four initial conditions we focus on here. Only the FPT distributions of koCP29 and koS-C show a slight shift to longer time. Interestingly, the dwell time distributions of the mutants (Suppl. Fig. 8) show much more variation than the FPT distributions.

For the trajectories with an FPT between 80 and 110 ps, the WT dwell time distribution (Suppl. Fig. 8a) shows that energy primarily stays in the D2 antennae in Monomer 2, but can travel to D1 antennae in Monomer 1 as well. The absence of CP29 causes the dwell time in the D1 antennae to increase, as the pathways to RC 2 are mostly interrupted. The dwell time in CP47 (Monomer 2) is shorter than in the WT and other mutants, but it is non-zero. This effect is most likely due to a single domain that is delocalized over CP29 and M-LHCII (C), which makes it impossible to completely disentangle the two subunits. While the exciton states in the domain disappear when they have more than 50% of contribution from pigments in CP29, some of the remaining excitons still have a contribution there and are spatially closer to CP47 (see Methods for more details). Additionally, in this FPT range, koS-C has an almost identical dwell time distribution to the WT, which confirms that S-LHCII (C) is more important for connecting with CP29 rather than M-LHCII, as mentioned earlier. On the other hand, the removal of M-LHCII (A) produces reduced dwell times for the D1 antennae, indicating again that M-LHCII (A) is connected to S-LHCII (B). Together they form pathways that are crucial for connecting S-LHCII of Monomer 1 and M-LHCII of Monomer 2.

For the trajectories with an FPT in the range 185 to 295 ps (Suppl. Fig. 8e-h), the dwell time distributions of the WT and all mutants are very similar to the corresponding distributions for the shorter FPT range (80 to 110 ps, Suppl. Fig. 8a-d). The most obvious difference is that the dwell times of D1 antennae are slightly longer on this timescale. Furthermore, the dwell time distributions of the WT, koCP29, and koS-C are all very similar. Only the removal of M-LHCII (A) blocks the pathways to the D1 antennae. This result suggests that initial excitations in M-LHCII (B) can access pathways that allow them to travel around the antenna system. Unlike the excitation of other subunits, these pathways have rather similar timescales, and therefore the dwell time distribution does not differ significantly for different FPT ranges.

Suppl. Note 7. Fast Transfer Pathways from CP43 to the RC

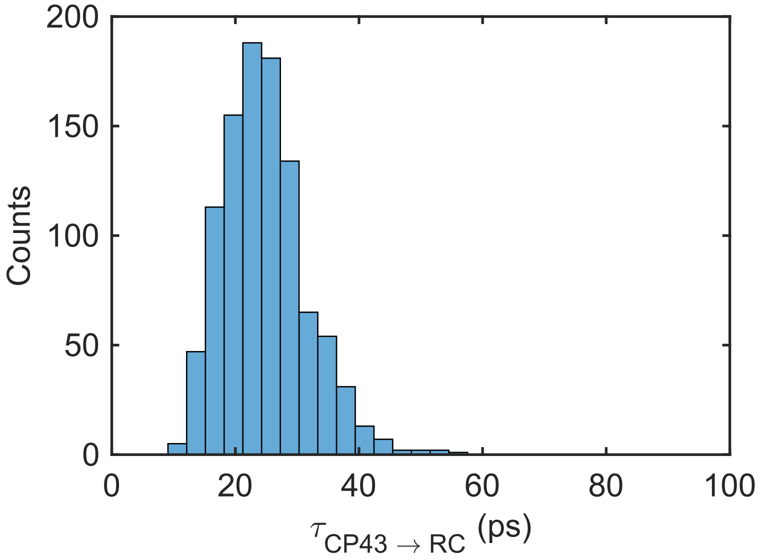

Suppl. Fig. 9 (a) Probability distribution of time constants for transfer from CP43 to RC at 300 K obtained from the kinetic model reported in this work. The distribution shares similar features as the distribution function for CP43 to RC transfer at 300 K reported in Figure 13 of Ref [4].

Figure 2b-c in the main text show distribution at short FPTs (on $\sim$ ps timescales). This is seemly contradictory with the CP43 to RC transfer reported in literature, which is typically in tens of ps timescales (40 ps in Ref [4] and 17 ps in Ref [1]). We note that these values reported in literature are the mean transfer rates from CP43 to the RC, averaged based on thermal population of the states within a domain/compartment. Particularly, Figure 13 in Ref [4] shows the distribution of these averaged rates over different inhomogeneous realizations (static disorder). This distribution shows how static disorder influences the transfer between CP43 to the RC, which is fundamentally different from the FPT distribution discussed in this work. The FPT distribution shows the different microscopic pathways present in the complex transfer network, as

illustrated in Figure 2a in the main text. In the case of CP43 to RC transfer, the FPT distribution shows that there are pathways much faster than the averaged rate. To compare with literature, we plot the distribution function according to the definition in the work of Raszewski and Renger [4]. Suppl. Fig. 9 shows the static-disordered rate distribution obtained from our model, which is similar to the one reported in Figure 13 of Ref [4]. Our model gives an inhomogeneously averaged transfer rate of $(25\text{ps})^{-1}$ compared to $(40\text{ps})^{-1}$ reported in Ref [4] and $(17\text{ps})^{-1}$ reported in Ref [1]. These values are of similar magnitude, and the small variations most likely originate from the different structures used in each model. In conclusion, no serious contradiction exists between our work and the results reported in the literature.

Supplementary References

[1] Bennett, D. I., Amarnath, K. & Fleming, G. R. A structure-based model of energy transfer reveals the principles of light harvesting in photosystem ii supercomplexes. *Journal of the American Chemical Society* **135**, 9164–9173 (2013).

[2] Leonardo, C. *et al.* Bidirectional energy flow in the photosystem ii supercomplex. *The Journal of Physical Chemistry B* **128**, 7941–7953 (2024). URL https://doi.org/10.1021/acs.jpcb.4c02508. PMID: 39140159.

[3] Raszewski, G., Saenger, W. & Renger, T. Theory of optical spectra of photosystem ii reaction centers: location of the triplet state and the identity of the primary electron donor. *Biophysical Journal* **88**, 986–998 (2005).

[4] Raszewski, G. & Renger, T. Light harvesting in photosystem ii core complexes is limited by the transfer to the trap: can the core complex turn into a photoprotective mode? *Journal of the American Chemical Society* **130**, 4431–4446 (2008).

[5] Müh, F., Madjet, M. E.-A. & Renger, T. Structure-based simulation of linear optical spectra of the cp43 core antenna of photosystem ii. *Photosynthesis research* **111**, 87–101 (2012).

[6] Mascoli, V., Novoderezhkin, V., Liguori, N., Xu, P. & Croce, R. Design principles of solar light harvesting in plants: Functional architecture of the monomeric antenna cp29. *Biochimica Et Biophysica Acta (BBA)-Bioenergetics* **1861**, 148156 (2020).

[7] Novoderezhkin, V., Marin, A. & van Grondelle, R. Intra-and inter-monomeric transfers in the light harvesting lhcii complex: the redfield–förster picture. *Physical Chemistry Chemical Physics* **13**, 17093–17103 (2011).

[8] Wei, X. *et al.* Structure of spinach photosystem ii–lhcii supercomplex at 3.2 å resolution. *Nature* **534**, 69–74 (2016).

[9] Su, X. *et al.* Structure and assembly mechanism of plant c2s2m2-type psii-lhcii supercomplex. *Science* **357**, 815–820 (2017).

[10] Woods, E. J., Kannan, D., Sharpe, D. J., Swinburne, T. D. & Wales, D. J. Analysing ill-conditioned markov chains. *Philosophical Transactions of the Royal Society A: Mathematical, Physical and Engineering Sciences* **381**, 20220245 (2023).

[11] Woods, E. J. & Wales, D. J. Analysis and interpretation of first passage time distributions featuring rare events. *Phys. Chem. Chem. Phys.* **26**, 1640–1657 (2024).

[12] Eyring, H. The activated complex and the absolute rate of chemical reactions. *Chem. Rev.* **17**, 65 (1935).

[13] Evans, M. G. & Polanyi, M. Some applications of the transition state method to the calculation of reaction velocities, especially in solution. *Trans. Faraday Soc.* **31**, 875 (1935).

[14] GMIN: A program for basin-hopping global optimisation, basin-sampling, and parallel tempering. http://www-wales.ch.cam.ac.uk/software.html (accessed on 4th August, 2022).

[15] Bortz, A. B., Kalos, M. H. & Lebowitz, J. L. A new algorithm for monte carlo simulation of ising spin systems. *J. Comput. Phys.* **17**, 10–18 (1975).

[16] Voter, A. F. Classically exact overlayer dynamics: Diffusion of rhodium clusters on rh(100). *Phys. Rev. B* **34**, 6819–6829 (1986).

[17] Fichthorn, K. A. & Weinberg, W. H. Theoretical foundations of dynamical monte carlo simulations. *J. Chem. Phys.* **95**, 1090–1096 (1991).

[18] Ruban, A. V. *et al.* Identification of a mechanism of photoprotective energy dissipation in higher plants. *Nature* **450**, 575–578 (2007).

[19] Ballottari, M., Mozzo, M., Girardon, J., Hienerwadel, R. & Bassi, R. Chlorophyll triplet quenching and photoprotection in the higher plant monomeric antenna protein lhcb5. *The Journal of Physical Chemistry B* **117**, 11337–11348 (2013).

[20] Park, S. *et al.* Chlorophyll-carotenoid excitation energy transfer in high-light-exposed thylakoid membranes investigated by snapshot transient absorption spectroscopy. *Journal of the American Chemical Society* **140**, 11965–11973 (2018).

[21] Son, M., Pinnola, A., Gordon, S. C., Bassi, R. & Schlau-Cohen, G. S. Observation of dissipative chlorophyll-to-carotenoid energy transfer in light-harvesting

complex ii in membrane nanodiscs. *Nature Communications* **11**, 1295 (2020).

[22] Hsieh, S.-T., Zhang, L., Ye, D.-W., Huang, X. & Cheng, Y.-C. A theoretical study on the dynamics of light harvesting in the dimeric photosystem ii core complex: Regulation and robustness of energy transfer pathways. *Faraday Discussions* **216**, 94–115 (2019).
